# Comparison of Therapeutic Effects of Omega-3 and Methylphenidate (Ritalin^®^) in Treating Children With Attention Deficit Hyperactivity Disorder

**Published:** 2014

**Authors:** Naser Dashti, Hoda Hekmat, Hamid Reza Soltani, Abolghasem Rahimdel, Mohammad Javaherchian

**Affiliations:** 1 Assistant Professor, Department of Psychiatry, School of Medicine, Yazd Branch, Islamic Azad University, Yazd, Iran.; 2 Resident, Department of General Surgery, School of Medicine, Qazvin University of Medical Sciences, Qazvin, Iran.; 3 General Practitioner, School of Medicine, Yazd Branch, Islamic Azad University, Yazd, Iran.; 4 Assistant Professor, Department of Neurology, School of Medicine, Shahid Sadoughi University of Medical Sciences and Health Services, Yazd, Iran

**Keywords:** Attention deficit hyperactivity disorder (ADHD), Methylphenidate, Omega-3, Ritalin^®^

## Abstract

**Objective:**

Attention deficit hyperactivity disorder (ADHD) is a fixed pattern of disregard and hyperactivity that is much more severe than what is normal in children of the same age. Multiple drugs are used for the treatment of children with ADHD; however, their side effects and efficacy are not clearly known. This study was designed to evaluate and compare the therapeutic effects of two drugs, that is, omega-3 and methylphenidate hydrochloride (Ritalin^®^), used to treat patients with ADHD.

**Methods:**

In a randomized, placebo control clinical trial in Yazd, Iran, 85 ADHD children were divided into two experimental and one control groups. Thus, 29 subjects were treated with Ritalin^®^, 28 subjects received omega-3, and the remaining 28 received placebo. The data collection tools used in this study consisted of the Conners' Parent Rating Scale and Teacher Rating Scale. The scores obtained from these questionnaires were analyzed using chi-square test and paired t-test in PASW Statistics.

**Results:**

The average age of the population was 8.22 (± 1.65) years. Significant associations were observed between Ritalin^®^ therapy and the changes before and after the treatment, and the omega-3 treatment and the changes before and after treatment (p < 0.001). There was no significant association between the placebo group and the changes before and after the treatment (p > 0.050). Omega-3 had considerable efficacy as well as Ritalin^®^ (P = 0.001).

**Conclusions:**

More attention should be given to screening, prevention, and treatment with omega-3 and its effective role in the development of the brain and mental health, and increasing children's attention span and thinking ability.

## Introduction

Attention deficit hyperactivity disorder (ADHD) is a fixed pattern of disregard and hyperactivity that is much more severe than what is normal for children of the same age group (1). Cognitive, functional, and hyperactivity are obvious aspects of this disorder (2, 3) and it is usually observed with other psychiatric disorders such as depression, anxiety, conduct and learning disorders (4, 5). Based on the presenting symptoms, ADHD can be divided into three subtypes—predominantly inattentive, predominantly hyperactive-impulsive, or combined if criteria for both types are met (6). The prevalence of this disorder has been reported to be 2-20% in the USA and also the prevalence of this disorder in boys is higher than girls (7). Stimulant drugs, such as methylphenidate, are used in the treatment of this disorder. About 75% of patients are responsive to this medicine. However, this medicine has possible risks, such as abuse potential, and complications such as insomnia, anorexia and even child growth complication. Moreover, an association has been observed between various behavioral disorders like ADHD and low plasma levels of long-chain unsaturated fatty acids especially eicosapentaenic acid/docosahexaenoic acid (DHA) (omega-3) in these patients. Therefore, investigating the effect of food supplements containing these fatty acids and comparing the results with methylphenidate (Ritalin^®^) treatment in these patients seems necessary. By increasing the plasma levels of these fatty acids, these patients' symptoms, academic performance, and quality of life are improved and the possible complication of methylphenidate (Ritalin^®^) are avoided (8). This study was designed to evaluate and compare the therapeutic effects of omega-3 and methylphenidate (Ritalin^®^) in treating patients with ADHD.

## Materials and Methods

This was a randomized placebo-controlled clinical trial performed using a parallel method on 85 children aged 6-12 years in Yazd (Tamin Ejtemaei Central Hospital), Iran, from April 2010 to March 2011. Conners' Parent Rating Scale and Teacher Rating Scale were used in this study. Children with a score of higher than 65 on both scales, IQ of higher than 70 using the Kaufman Brief Intelligence Test, and whose parents gave an informed consent were included. However, children with occurrence of unintended side effect of methylphenidate, who had previous treatment for ADHD, who had taken omega-3 supplement during the previous 3 months, and who had severe psychiatric disorders were excluded from the study. The questionnaires were completed and collected by parents; then, the questionnaires were reviewed and evaluated by researchers to obtain the opinion of parents on the existence of the disorder in their children. The Teacher Rating Scale was given to the children's school to ask their teacher's opinion, and then, the teachers forms were reviewed and evaluated by researchers. Children who had ADHD based on both questionnaires were introduced to professionals for treatment. To be categorized as ADHD, the child must have at least 6 signs of attention deficit or at least 6 symptoms of hyperactivity and impulsivity, or both based on the DSM-IV criteria. Moreover, individual score from each questionnaire should be equal to or greater than 12 and the overall score of each subject from the total of both questionnaires should be equal to or greater than 24. In the present study, cases that only showed signs of attention deficit in both questionnaires were considered mainly as a type of inattentiveness. Cases which only showed signs of hyperactivity-impulsivity in both questionnaires were considered mainly as a type of hyperactivity-impulsivity. In addition, cases which showed signs of hyperactivity-impulsivity and attention deficit symptoms in both questionnaires were considered as a combined type. Thus, 29 patients were treated with Ritalin^®^ and 28 patients with omega-3, and the remaining 28 received placebos. Patients were divided into 3 groups using even and odds table. The first 29 patients were treated with therapeutic doses (0.3-1 mg/kg) of Ritalin^®^ 3 times a day, and the second 28 patients with therapeutic doses of omega-3 (1 g), one capsule per day for 3 days. The third group received placebo as capsules similar to the omega-3 group). The results of treatment were evaluated after 2 and 4 weeks, and again at the end of the treatment. The questionnaires were completed by the parents and the teacher of the treated subject and the scores were compared with that of questionnaires completed prior to the treatment. The scores obtained from these questionnaires were analyzed using the chi-square test and paired t-test in PASW Statistics, (version 18.0; SPSS Inc., Chicago, IL, USA).

## Results

In this study, of the 29 patients in the Ritalin^®^ therapy group, 58.6% were males and 41.4% females (17 boys and 12 girls). Of the 28 patients in omega-3 therapy group, 46.4% were males and 53.6% females (13 boys and 15 girls). Moreover, of the 28 patients in the placebo group, 64.3% were males and 35.7% females (18 boys and 10 girls). According to the obtained results, there was no statistically significant difference between the frequency of sex in the 3 groups (p = 0.387). Among the 85 patients, 20 patients had attention deficit disorder (ADD), 21 subjects had hyperactivity-impulsivity, and 44 were mixed (combined) type. There was no statistically significant difference between the frequency of the disease types in the three treatment groups (p = 0.595). The average age of the target population was 8.22 (±1.65) years. The mean scores (±standard deviations) before the treatment were 24.20 (± 9.76) in the Ritalin^®^ group, 25.89 (± 9.96) in the omega-3 group, and 22.58 (± 8.52) in the placebo group. Pre-treatment scores did not significantly differ in these three groups, using ANOVA statistical test (P = 0.126). In addition, the mean score (± standard deviation) after treatment was 17.81 (± 10.32) in the Ritalin^®^ group, 18.44 (± 8.36) in the omega-3 group, and 22.66 (± 8.01) in the placebo group. Paired t-test showed a statistically significant difference in the Ritalin^®^ group before and after treatment (p = 0.001). It also showed a statistically significant difference between treatment with omega-3 before and after the treatment. Nevertheless, paired t-test, based on a p value of 0.888, showed no statistically significant difference between treatment with placebo before and after the treatment (Table 1). On the other hand, using paired t-test and p > 0.050, the efficacy of the 2 examined drugs was significantly higher than placebo (Table 6). However, the changes of hyperactivity-impulsivity type in the Ritalin^®^ and omega-3 groups were significant. As a result, a statistically significant association was found between treatment with the first two intended drugs and changes before and after the treatment in the hyperactivity-impulsivity type. A statistically significant association was also observed between the placebo group and the changes before and after the treatment in the hyperactivity-impulsivity type (Table 3). In assessing the mean scores of combined type and its changes in the three study groups (before and after the treatment) with paired t-test, P-values of 0.001, 0.001, and 0.290 were obtained in the three groups, respectively. The mean score of combined type and its changes were statistically significant in the Ritalin^®^ and omega-3 groups. Thus, a statistically significant association was observed between treatment with the first two intended drugs and changes before and after the treatment in combined type. Nevertheless, there was no statistically significant difference between the changes at the beginning and end of our study in the combined type in the placebo group (Table 4). 

**Table 1:** Average scores of disease and its changes in the three study groups, before and after the treatment

**Group**	**Number**	**Pre-treatment scores**	**Post-treatment scores**	**Changes**	**P-value** *
		**Mean ± SD** †	**Mean ± SD**	**Mean ± SD**	
**Ritalin** ^®^	29	24.20 ± 9.76	17.81 ± 10.32	6.39 ± 5.39	0.001
**Omega-3**	28	25.89 ± 9.96	18.44 ± 8.31	7.44 ± 6.80	0.001
**Placebo**	28	22.58 ± 8.52	22.66 ± 8.01	-0.07 ± 2.66	0.888

* ANOVA;

† Standard deviation

**Table 2:** Average scores of ADD type and its changes in the three study groups, before and after the treatment

**Group**	**Number**	**ADD** †			
		**Pre-treatment scores**	**Post-treatment scores**	**Changes**	**P-value** *
		**Mean ± SD** ‡	**Mean ± SD**	**Mean ± SD**	
**Ritalin** ^®^	7	16.85 ± 3.83	4.77 ± 1.80	2.71 ± 3.30	0.073
**Omega-3**	4	15.50 ± 1.77	13.12 ± 2.25	2.37 ± 3.70	0.290
**Placebo**	9	15.11 ± 1.13	15.55 ± 2.74	-0.44 ± 2.77	0.645

* ANOVA;

† Attention deficit disorder;

‡ Standard deviation

**Table 3:** Average scores of hyperactivity-impulsivity type and its changes in the three study groups, before and after the treatment

**Group**	**Number**	**Hyperactivity-impulsivity**			
		**Pre-treatment scores**	**Post-treatment scores**	**Changes**	**P-value** *
		**Mean ± SD** †	**Mean ± SD**	**Mean ± SD**	
**Ritalin** ^®^	8	17.25 ± 2.72	08.37 ± 4.35	-8.87 ± 1.16	0.005
**Omega-3**	7	18.28 ± 2.82	12.28 ± 4.36	-6.00 ± 3.14	0.002
**Placebo**	6	15.58 ± 1.46	17.16 ± 1.29	-1.58 ± 0.86	0.006

* ANOVA;

† Standard deviation

**Table 4:** Average scores of combined type and its changes in the three study groups, before and after the treatment

**Group**	**Number**	**Combined type**			
		**Pre-treatment scores**	**Post-treatment scores**	**Changes**	**P-value** *
		**Mean ± SD** †	**Mean ± SD**	**Mean ± SD**	
**Ritalin** ^®^	14	31.85 ± 8.51	25.30 ± 9.52	6.82 ± 5.09	0.001
**Omega-3**	17	31.47 ± 8.86	22.23 ± 8.42	9.23 ± 7.67	0.001
**Placebo**	13	31.00 ± 4.33	30.11 ± 5.05	0.88 ± 2.88	0.290

* ANOVA;

† Standard deviation

**Table 5:** Comparison of rate of disease score changes in the three study groups

**Group**	**Number**	**Average**	**SD** †	**MIN**	**MAX**
**Ritalin** ^®^	29	-6.39	5.39	-2.5	17
**Omega-3**	28	-7.44	6.70	-6.0	29
**Placebo**	28	-0.07	2.66	-3.0	06
**Total**	85	-4.61	6.12	-3.0	29

† Standard deviation

**Table 6:** Binary comparison of rate of score changes due to treatment in the three groups

**Groups compared**		**Average changes**	**P-value** *
**Ritalin** ^®^	Omega-3	-1.04	0.449
**Ritalin** ^®^	Placebo	-6.46	0.001
**Omega-3**	Placebo	-7.51	0.001

* ANOVA

Moreover, using the analysis of variance (ANOVA) test, significant difference was obtained between disease score changes in the three study groups (p = 0.001) (Table 5). Furthermore, a statistically significant difference was found in the analysis of treatment groups mutually between the placebo and Ritalin^®^ and also placebo and omega-3. However, no statistically significant difference was observed between the average of the changes of scores in the two groups, which were treated with Ritalin^®^ and omega-3 (p = 0.449) (Table 6).

## Discussion

The main objective of this research was to review and compare the therapeutic effects of Ritalin^®^, omega-3, and placebo in the treatment of ADHD. As our findings showed, no statistically significant difference was found between omega-3 and Ritalin treatment. A statistically significant difference was found between the two drugs and recovery changes in subtypes of hyperactivity-impulsivity and combined type. However, there was no statistically significant difference between recovery changes in the placebo group in these two subtypes. A similar study was performed to evaluate the effects of omega-3 at high doses in ADHD patients. At the end of 8 weeks of study on 9 children of 8-12 years of age (6 boys and 3 girls), a reduction was observed in the symptoms of this disorder (9). In a similar study which was performed in Japan in 2004, the children were randomly divided into two groups, one group was treated with DHA and the other was the control group. No statistically significant difference was observed in the 2 groups at the beginning and end of the 8 weeks (10). In a study performed in London in 2005, about 40% of the children with ADHD had fatty acid deficiency symptoms after laboratory study, but only 9% in the control group had this problem (10). In addition, Richardson and Puri have written in their reports about the reduction of symptoms and behavioral problems following treatment with supplements containing fatty acids (11). Previously, the effects of similar compounds, such as fish oil, have been investigated; however, it was only found to strengthen the short-term memory without any change in patients' ADHD symptoms (10). Furthermore, in another study in the USA, the symptoms improved in Ritalin^®^ users compared with a placebo group (12). 

## Conclusion

The findings of the present study showed the effective role of omega-3 in treating patients with ADHD. Thus, more attention should be given to screening, prevention, and treatment with omega-3 and its effective role in the development of the brain and mental health, and increasing children's attention span and thinking ability. 

***Limitations***

Optimum accuracy was not attainable because parents and teachers had to complete the questionnaires individually. Moreover, the duration of our study was longer than expected due to the drop-out rate. Incomplete questionnaires were the main reason for this drop-out rate. 

## Notes

**Declaration of interest:** None.

## Notes

**Clinical Trial Registration-URL: **http://www.irct.ir. IRCT Code: IRCT201304035393N3.
